# Bicycle Facilities Safest from Crime and Crashes: Perceptions of Residents Familiar with Higher Crime/Lower Income Neighborhoods in Boston

**DOI:** 10.3390/ijerph16030484

**Published:** 2019-02-07

**Authors:** Anne C. Lusk, Walter C. Willett, Vivien Morris, Christopher Byner, Yanping Li

**Affiliations:** 1 Department of Nutrition, Harvard T. H. Chan School of Public Health, Boston, MA 02115, USA; wwillett@hsph.harvard.edu (W.C.W.); yanping@hsph.harvard.edu (Y.L.); 2 Mattapan Food and Fitness Coalition, Boston, MA 02126, USA; vivien.morris@gmail.com; 3 Boston Centers for Youth & Families, Boston, MA 02120, USA; christopher.byner@boston.gov

**Keywords:** bicycle, low income, ethnic-minority, crime, crash, cycle tracks

## Abstract

While studies of bicyclist’s perceptions of crime and crash safety exist, it is also important to ask lower-income predominantly-minority residents what bicycle-route surface or context they perceive as safest from crime and crashes. With their insights, their chosen bike environments could be in engineering guidelines and built in their neighborhoods to improve residents’ health and lessen their risk of exposure to crime or crashing. This study involved two populations in Boston: (a) community-sense participants (eight groups-church/YMCA *n* = 116); and (b) street-sense participants (five groups-halfway house/homeless shelter/gang members *n* = 96). Participants ranked and described what they saw in 32 photographs of six types of bicycle environments. Quantitative data (Likert Scale 0–6 with 0 being low risk of crime/crash) involved regression analysis to test differences. Qualitative comments were categorized into 55 themes for surface or context and if high or low in association with crime or crashes. For crime, two-way cycle tracks had a significantly lower score (safest) than all others (2.35; *p* < 0.01) and share-use paths had a significantly higher score (least safe) (3.39; *p* < 0.01). For crashes, participants rated shared-use paths as safest (1.17) followed by two-way cycle tracks (1.68), one-way cycle tracks (2.95), bike lanes (4.06), sharrows (4.17), and roads (4.58), with a significant difference for any two groups (*p* < 0.01) except between bike lane and sharrow (*p* = 0.9). Street-sense participants ranked all, except shared-use paths, higher for crime and crash. For surface, wide two-way cycle tracks with freshly painted lines, stencils, and arrows were low risk for crime and a cycle track’s median, red color, stencils, and arrows low risk for crash. For context, clean signs, balconies, cafes, street lights, no cuts between buildings, and flowers were low risk for crime and witnesses, little traffic, and bike signals low risk for crash. As bicycle design guidelines and general Crime Perception Through Environmental Design (CPTED) principles do not include these details, perhaps new guidelines could be written.

## 1. Introduction

Bicycling should be enabled for all populations because of the positive associations with weight control [1,2,3] improved cardiac function [4], overall health [5], and lower mortality [6,7] but, in the U.S., only 0.6% of the population 16 and over commutes regularly by bicycle [8]. Residents in lower-income ethnic-minority neighborhoods may be hesitant to bike due to the environments through which they would ride. Providing safe bicycle facilities in these neighborhoods [9,10] may help address a racial injustice because African-American and Hispanic populations engage in less physical activity [11,12] and have higher rates of obesity [13]. If African American and Hispanic populations were asked to select the bicycle environments they prefer and their chosen environments were included in the bicycle design guidelines and then built, chances would be greater that they would bicycle more. 

For crime, residents in lower-income African American and Hispanic communities may engage in less physical activity, including not bicycling [14], due to perceptions of crime [15,16,17,18]. If bicyclists are aware that violent crimes have been committed along a bike route, they may select other means of travel [19]. Perception of crime can be lessened, as identified by Jacobs through “eyes of the street”[20], Jeffrey through Crime Prevention Through Environmental Design (CPTED) [21,22], Newman through defensible space [23], and Kelling and Coles through “Fixing Broken Windows” [24]. Their proposed changes to the built environment can be implemented but perhaps crime-related improvements would be different if for bicycle environments and if identified by residents in lower-income ethnically diverse communities. 

For crashes, willingness to bicycle is based on the perception of crash risk [25]. African-American and Hispanic bicyclists already experience higher rates of car/bicycle crashes compared with White bicyclists [9,26]. For bicyclists in the U.S., the sanctioned practice has involved sharing the road with cars [27,28], which requires lane-command and enrolling in classes to learn how to operate the bicycle as a vehicle [29]. The crash rate of bicyclists in the United States is 3.75 per million km bicycled compared to 0.14 in the Netherlands [30,31], due in large part to the Dutch having 29,000 km of cycle tracks (barrier-protected, bicycle-exclusive facilities beside sidewalks) [32] and the U.S. having only 30 km [33]. Research has suggested that cycle tracks are safer [33,34,35,36,37,38,39] and preferred [40], including being preferred by lower-income ethnic-minority residents [41]. By May 2017 in the U.S., the number of cycle tracks had increased to 406 but the cycle tracks are isolated and only average 1.2 km in length [42]. In 10 cities in the U.S. that had installed safer bike facilities, fatalities and severe injuries of bicyclists per 100,000 trips had declined between 43% to 79% [43]. Even though the new bike facilities in those 10 cities reduced cyclists’ fatalities and injuries, safe bicycle facility networks continue to not be built in all communities [36].

For help in designing the best bike facilities, U.S, guidelines are consequential because state engineers approve funding for and build facilities following these guidelines. The authors of the early bicycle guidelines were male engineers and their recommendations remained unchanged for years as they cut and pasted the same text for subsequent guideline editions [33,44]. The early authors were also skilled male bicyclists who preferred to operate their bicycle as a vehicle. Unlike male and female bicyclists who pedal at different speeds [45], drivers move the vehicle with an accelerator pedal, eliminating strength-in-driver differences. The automobile also ensures the safety of the occupants with features such as roll bars, seat belts, and air bags [46]. The highway environment protects the most vulnerable drivers, including a drowsy driver who awakens from the sound of a rumble strip [47]. In contrast, the bicycle environment assumes bicyclists possess the maximum skill set for bicycling and that bicyclists have no human flaws such as being distracted. There are also gender and crime apprehension differences. The concept of “fight or flight” in human behavior science is well known but the rats used in the experiment were male. If female rats are put in the same environment, the female rats “tend and befriend” because they cannot, like the males, fight or flee as effectively [48]. 

While the practice in the U.S. has been to build bicycle facilities based primarily on the perceptions of educated male engineers [33] according to the American Community Survey (2008–2012), the majority who use the bicycle as their transportation to work earn less than $10,000 [49]. These lower-income bicyclists might not always be asked which bicycle environments they would prefer and why. Therefore, this research would first learn from lower-income ethnic-minority residents which bicycle environments, as shown on a large screen, make them feel more or less vulnerable to an act of crime and more or less likely to hit by a vehicle. Their perceptions would also be compared based on gender, age, if they could ride a bicycle or not, and if they were in the community-sense groups (church/YMCA) or the street-sense groups (halfway house/homeless shelter/gang members). Second, the study would identify which bicycle environment surface and surrounding three-dimensional context elements make the resident feel more or less vulnerable to crime or crash. Third, the participant’s perceptions about the bicycle environments would be aligned with the basic tenants in bicycle design guidelines and crime theories to determine if there were different insights from lower-income ethnic-minority populations. The findings could perhaps be incorporated in new bicycle design guidelines or CPTED principles to improve all bicyclists’ safety from crime and crash. 

## 2. Materials and Methods

The methods included survey locations and groups, theory, survey, and data analysis. 

### 2.1. Survey Locations and Groups

The survey was conducted in or near Roxbury, Mattapan, and Dorchester as these areas have, compared to other areas in Boston, historically been under-resourced and had a history of higher crime [50] and lower income [51,52]. Crime rates have dropped over the years but the perception of crime about a community can linger both in and outside a community. These neighborhoods once had streetcars and, with the introduction of cars, many streets are now one-way with cars parked on both sides. U.S. Census data from 2010 reported that these three neighborhoods had higher densities of predominantly Black and other minority populations (Roxbury—41.4% Black and 27.0% Latino; North Dorchester—44.0% Black and 22.6% Latino; South Dorchester—45.8% Black and 14.7% Latino; Mattapan—80.4% Black and 11.7% Latino) [53]. Groups contacted had members with a high community sense (churches, YMCA’s, etc.) or a high street sense (halfway houses, homeless shelters, gang connections, etc.). Street-sense groups were included because individuals who have committed crimes or know of crime opportunities provide valuable insights [54,55]. 

Calls to ninety-one organizations generated lists of potential groups. Groups were then emailed the survey, information, and flyer to help recruit attendees to the dinner and survey. A portable LCD projector and a large screen allowed for showing the slides in different locations chosen by the residents, e.g., their own church, halfway house, or homeless shelter. The groups were small (no more than 30 per group) and often part of regularly scheduled meetings. To demonstrate respect and thanks, full dinners or desserts and coffee came from a well-regarded catering establishment with staff of under-employed men and women in the Boston area. 

### 2.2. Theory

This study aligns with the ecological model that suggests the environmental setting, plus individual and social factors, can foster well-being [56]. To offer a hierarchy to the ecological model, Maslow’s pyramid was applied, i.e., only after basic needs are met can higher needs be met [57]. This study specifically applied the Maslow transportation Level of Service (LOS) [58] i.e., before time, societal acceptance, cost, comfort, and convenience needs of bicyclists are met, the basic needs of security (from crime) and safety (from crash) must be met. The study is also framed on Crime Prevention Through Environmental Design (CPTED) principles, as posited by Jacobs [20], Jeffrey [21], Newman [23], and Kelling and Coles [24]. 

### 2.3. Survey

In structured presentations with feedback, participants were asked if they would rate (Likert Scale 0–6 with 0 being low risk of crime/crash) and comment on the variety of pictures of bicycle environments. Colored pictures were shown on a screen because no higher crime/lower income neighborhood in the U.S. contains all the state-of-art bicycle facilities. Participants were given the paper survey and a pencil and provided with instruction on the survey and verbally. 

**Figure 1:**
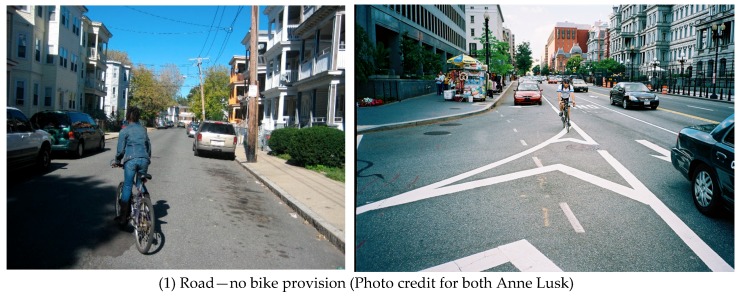

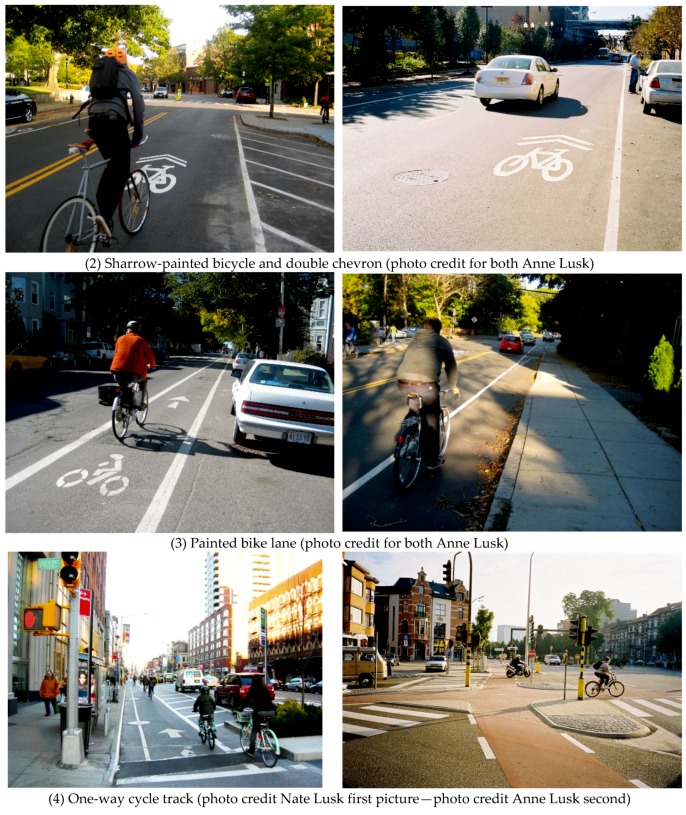

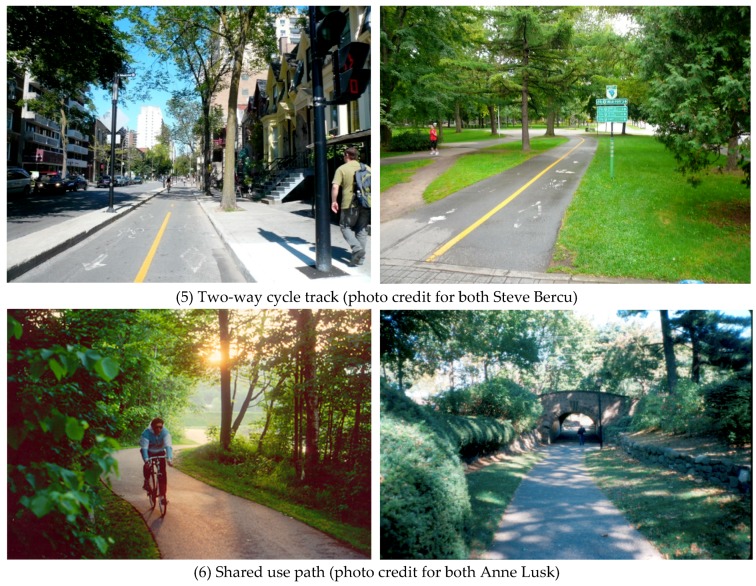
Two pictures of each of the facility types included in the survey of 32 slides.

Over 100 photographs of six different types of bicycle facilities were assembled including: (1) roads with no bicycle provisions; (2) roads with sharrows (bicycle stencil with a double chevron); (3) painted bicycle lanes; (4) one-way cycle tracks; (5) two-way cycle tracks; and (6) shared-use paths (Table 1 and Figure 1). The photographs all had good daylight, no or only a few bicyclists present, and a dominant view of the bicycle provision. Because so few cycle tracks exist in the U.S., some generic photographs were from Montreal and cities in Western Europe. Photographs of the same facility varied based on width, paint, road-separation treatment, stencils, trees, and context. A City of Boston Official, who had an understanding of bike facilities best understood by residents in Roxbury, Mattapan, and Dorchester, reviewed and helped select the photographs. In total, 32 photographs served as representations of the different bicycle facilities. 

For quantitative data, the paper survey asked respondents to look at the pictures shown on the large screen and mark their answer to the question, “Do you feel the chance of crime is low or high (0–6 with 0 being low chance of crime/crash) if you bicycle in this place?” The participants were told that crime meant being fearful that some negative occurrence might happen to them as a bicyclist in that location. For crash, the question was repeated and participants were told that crash meant being fearful that a vehicle might hit them. Participants could also volunteer their age, gender, and if they knew how to bicycle. 

For qualitative data, the pictures, when shown again, gave participants the opportunity to say what they saw in the picture that might be associated with low or high perceptions of crime and crashes. The participants in each of group rated the 32 pictures in about 1/2 hour followed by an additional 1/2 hour for discussions about each picture. The study received an Institutional Review Board (IRB) exemption as participants completed the surveys voluntarily and anonymously. 

### 2.4. Data Analysis

The different perceptions of each bicycle environment picture were analyzed based on male or female, age, if they could ride a bicycle or not, and if they were in the community-sense groups (church/YMCA) or the street-sense groups (halfway house/homeless shelter/gang members). Demographics identified differences between the “community sense” and “street sense” participants. The mean scores (95% confidence intervals) for perception of crime and crash risks for each of the 6 bicycle facility types were adjusted for age, sex, whether they know how to bicycle, and street sense or community sense using general linear regression models, and were compared using Tukey’s multiple comparisons tests. A figure was prepared that separately displayed the crime and crash perception for each of the six bicycle facilities. Two models, with one for the crime score and the other for the crash score as the dependent variable, provided the rated score of each bicycle facility type. Regression analysis of general linear models (GLM) were used to test the difference between groups based on sex, age, ability to bicycle, group type, and bicycle facility type with mutual adjustment for each other, separately for the crime score and the crash score. 

For analysis of the qualitative comments, specialized software, such as nvivo, was not utilized because each picture had to be viewed and understood for data within that picture to understand the intent of the qualitative comment about that picture. The qualitative comments on the transcript about each picture received codes while looking at that picture. The categorization for each comment was according to whether the comment related to the surface/bicyclist’s right-of-way or the three-dimensional context and with high/low crime and crash categories for each. The codes totaled 72 types of responses but if only one individual made a comment, it was not included in the table, resulting in 55 codes. Frequency was determined for the number of times the 493 qualitative comments were mentioned in any of the structured presentations. 

The findings from the qualitative comments were compared with recommendations in the bicycle design guidelines and CPTED to determine if the participant’s perceptions were reflected in standards for building bicycle facilities. If there were dissimilarities, the recommendation could be to write design guidelines and develop crime prevention principles based on the perceptions of ethnically diverse lower-income individuals and not based only on the perceptions of white male engineers, the principle authors of the past bicycle guidelines. 

## 3. Results

Two hundred and twelve individuals in thirteen structured-presentation groups completed the surveys. Eight of the groups were strong community sense (church/YMCA *n* = 116) and five were strong street sense (gang/halfway/homeless *n* = 96) (Table 2). The group sizes ranged from 9 to 28 participants with a mean age of 36, 42% female, and 87% knowing how to bicycle. Twelve of the groups were comprised primarily of African American individuals with a mixed population in one homeless shelter.

### 3.1. Crime Perception Quantitative Data

**Figure 2:**
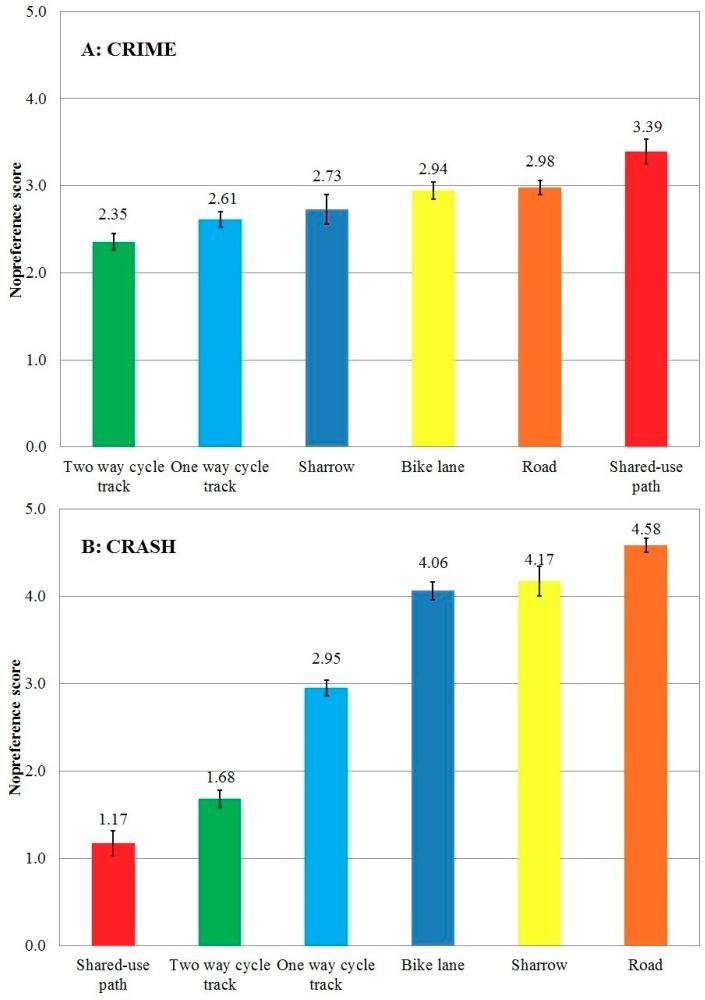
Adjusted mean scores (95% confidence intervals) for perception of crime and crash risks for the 6 bicycle facility types. Adjusted for age, sex, whether they know how to bicycle, and street sense or community sense using general linear regression models. Tukey’s multiple comparisons tests were used to compare the differences between each two groups.

All of the bicycle facilities were rated for crime (mean scores = 2.35–3.39) (0–6 with 0 being low chance of crime/crash). Two-way cycle tracks (rated score = 2.35, 95% confident interval (CI): 2.26–2.45) had a significantly lower score for crime (safer) than all others (Ps < 0.01) and share-use paths (3.39 (3.25–3.53)) had a significantly higher score (less safe) (Ps < 0.01) (Figure 2A). For crime perceptions by population, gender differences were statistically significant for roads (2.91 males/3.07 females), shared-use paths (3.14 males/3.68 females), and bike lanes (2.85 males/3.04 females) (*p* < 0.05) (Table 3). Two-way cycle tracks were safest from crime and perceived equally by males and females. For all bicycle facilities together, females perceived crime higher (*p* < 0.0001). For the two-way cycle track, participants who cannot ride a bike had the highest perception of safety from crime (2.19). Differences were statistically significant for street sense individuals who, compared with community sense individuals, rated all of the bicycle facilities, except shared-use paths, higher for crime perception. 

### 3.2. Crash Perception Quantitative Data

For crashes, participants rated shared-use paths as safest (1.17 (1.02–1.31)), followed by two-way cycle tracks (1.68 (1.58–1.78)), one-way cycle tracks (2.95 (2.86–3.04)), bike lanes (4.06 (3.96–4.16)), sharrows (4.17 (4.00–4.34)), and roads (4.58 (4.50–4.66)), with a significant difference for any two groups (Ps < 0.01) except between bike lane and sharrow (*p* = 0.9) (Figure 2B). For crash perceptions by population, gender differences were statistically significant for roads (4.38 males/4.85 females), shared-use paths (1.30 males/0.99 females), bike lanes (3.89 males/4.26 females), and sharrows (3.95 males/4.44 females) (*p* < 0.05) (Table 4). Of all the populations and all the bike facilities, females perceived the shared use path as safest from crashes with vehicles (0.99). Street sense participants rated all the bicycle facilities, except the shared use path, as more dangerous for vehicle/bicycle crash. 

### 3.3. Crime Perception Qualitative Comments

The categorized qualitative comments totaled for crime include: a) surface/bicyclist’s right-of-way—high/low risk of crime; and b) three-dimensional context—high/low risk of crime (Table 5). For surface/bicyclist’s right-of-way, participants perceived high crime risk with narrowness of cycle track, faded bike-lanes, and old bike symbols. Participants perceived low crime risk with a two-way cycle track because the bicyclist knew how to get back home. For context in the surrounding area, participants perceived high crime risk if there were few to no people around, no lights, dense trees/bushes/high grass, hiding spots/cuts between buildings, lack of maintenance, dense crowds, and lots of parked cars or cars being driven. Low crime risk was associated with many people being out (as with sidewalk cafés), cleanliness, nice signs, no dark alleys, balconies on houses, light, flowers, limbed-up trees, high-end stores, and lots of little shops.

### 3.4. Crash Perception Qualitative Comment

The categorized qualitative comments totaled for crash include: a) surface/bicyclist’s right-of-way—high/low risk of crash; and b) three-dimensional context—high/low risk of crash (Table 5). For the surface/bicyclist’s right-of-way, participants perceived high crash risk with a sharrow near the middle of the road, a narrow right-of way, a narrow cycle track, no bike signs/stencils, or no space between parallel-parked cars and a one-way cycle track into which car doors would open. For surface right-of-way, participants perceived low crash risk with island barriers between the road and the cycle track, a red cycle track, a wide right-of-way, bike symbol/stencil/arrows, and a one-way cycle track. For the context in the surrounding area, participants perceived high crash risk with many drivers, close cars, painted bike lanes near opening car doors, proximity to bus stops, sharing the road with buses, high vehicle speeds, and confusing paint. For context, participants perceived low crash risk when there were more people around, not a lot of car traffic, and a bike signal at the intersection.

### 3.5. If the Findings Are in Bicycle Facility Design Guidelines and CPTED Principles

In comparing the preferences from this study with the American Association of State Highway and Transportation Officials (AASHTO) 2012 “Guide for the Development of Bicycle Facilities,” the current AASHTO guide does not include cycle tracks [27]. The guideline does include one brief paragraph about aesthetics and suggests that trees make riding cooler and provide a windbreak and that bicyclists prefer to be near shopping districts and have a view. The AASHTO bike guideline recommends studying crash reports to learn where to make improvements but that would be after and not before a crash. The National Association of City Transportation Officials (NACTO) bicycle guideline [59] mentions the need to design for safety from crash but only recommends building certain types of bicycle facilities, such as cycle tracks. Low stress routes away from high traffic areas and one-way closed-to-through-traffic bike routes are recommended [60] but these would be in areas with fewer people. 

A comparison between the populations who provided the data for this study and Jane Jacobs reveals differences. Jacobs proposed lowering crime through “eyes on the street” in her 1961 book “The Life and Death of Great American Cities”[20]. She wrote about her neighborhood in Greenwich Village in New York City where her family and others had purchased three-story affordable historic housing to restore [61]. Her neighborhood was set for demolition as part of urban renewal but an official, after seeing the homes’ high ceilings and grand fireplaces, determined that the residents were “clearly not slum dwellers.” Although Jacobs rode a bicycle to her job as editor at Architectural Forum, her writings only encouraged people to see the city while walking [62]. The streets in the West Village around 1958 included wide sidewalks, parallel parking, and roads for cars with no provisions for bicyclists. Her suggestions about “eyes on the street” would have deterred crime but her community sidewalks and streets were to remain the same and she, a daughter of a doctor in a neighborhood deemed not a slum, may have been less aware of which environmental features best deter crime. 

There also are differences between the findings in this study and Crime Prevention Through Environmental Design (CPTED) principles. CPTED proposes having people around for surveillance and also not having dense shrubbery behind which a criminal could hide [20,21,23]. Four principles have guided CPTED: (1) territoriality; (2) natural surveillance; (3) activity support; and (4) access control [63]. Though territoriality might work for a building or a street in a gated residential compound, bicycle facilities are public transportation throughways. Natural surveillance, activity support, and access control involve high participation by local residents and, especially on a Main Street with shops and cafes, all individuals should be welcomed. Crime is complex, especially as crime against a person and the crime of household robbery are different [64]. Crime against a bicyclist involves the rider, a person, or their bicycle, property, and both benefit from design guidelines written to serve the most vulnerable populations. 

## 4. Discussion

A bicyclist’s fear of crashing is a concern everywhere but in higher crime/lower income neighborhoods the risks of having someone steal the bike while riding or being attacked at night in a park lessens willingness to bike. Participants thought the two-way cycle track the safest from crime because then the bicyclist knew how to get back home, a necessary feature in neighborhoods with many one-way streets. To deter crime and crashes, participants perceived that cycle tracks should be wide and freshly painted with a red surface color, bike symbols, and directional arrows. The cycle tracks should also have a median, be on a main street, be near nice shops/mixed residential, not pass by fast food places/low end stores/warehouses, have street lights, not have dense bushes/trees, be clean (no litter), have nearby flowers and plants, and have a bike signal. For safety from crash, participants thought the shared-use path and two-way cycle track were both safe but the shared-use path was too isolated to deter crime. 

The participants also commented on things that cannot be immediately changed including oil on the road (cars not well maintained), low-end shops, hiding spots and cuts (spaces between buildings), unpainted front stoops, leaning telephone poles, too few people around, drivers moving fast in multiple directions, and too many intersections coming together. Therefore, the focus should be on built environment features that are changeable but also additionally beneficial. Gentle guidance could direct bicyclists in lower income/higher crime neighborhoods where to safely bike (cycle tracks with a painted bike surface and a median), which direction to bike (bike stencils with directional arrows), and when to cross an intersection (bike signals). Adding these directional bike features would lessen their risk of being ticketed [65,66] and help with way-finding [67]. 

### 4.1. Crime Perception

Participants identified design solutions for crime but the issue would be whether to install the cycle track first or lower crime first. The Dutch have safe bike environments on virtually all of the streets but, in the U.S., the lower income neighborhoods are the last to receive safe bike environments [41,68,69]. Though bicyclists prefer what are called “low stress routes,” or routes that do not expose bicyclist to high levels of stress from vehicular traffic [70], a quiet side street with few people is isolated. A better policy might be to build cycle tracks first but on the streets with the lowest crime and highest visibility, as on Main Street. Neighborhoods could have new-to-the-community cycle tracks as “cues to care” [71] that display personal caring and good human intention. Having many bicyclists would make the neighborhood social [72] because, unlike car occupants, bicyclists talk to neighbors. 

While trees were associated with high crime, sight lines and lighting were associated with low crime. Large trees obscure lighting from tall cobra-head streetlights but cycle-track-directed lower lighting could brighten the bike route while leaving the overhead canopy for shade. Parallel parked cars were associated with a high risk of crime and parked cars by a bike lane increase crashes [73]. Not having parallel-parked cars on both sides and having a cycle track on one side might lower crime and crashes. Therefore, giving free parking permits and fostering road car storage [74] should be re-assessed with metrics of environment, health, and equity in a new Level of Service (LOS) [75]. Cycle tracks in Denmark provide transportation equally to the poorest and wealthiest, better guaranteeing that the poorest will not stay poor [76]. In the Maslow transportation Level of Service (LOS), safety and security were basic needs to be met first [58] because risks make people unwilling to be active [18,77]. Women were more concerned about crime biking on the road, shared-use path, or bike lane than males, a factor perhaps attributed to evolution, female’s risk aversion, and the need to care for young [48]. 

This study involved individuals who knew crime and similar survey research had been conducted on bus and bus stop designs related to perceptions of crime in homeless shelters in inner city Detroit [78]. Those qualitative comments informed the design of new buses and this research might have useful information to include in bicycle design guidelines, resulting in equitable bicycle facilities.

### 4.2. Crash Perception

Crash risk was lowest with shared-use paths and two-way cycle tracks yet males perceived a shared-use path somewhat less safe from crashes. The males may already know of the conflicts with other recreationists on shared-use paths [79,80]. For crashes, females judged roads, shared-use paths, bike lanes, and sharrows as less safe, as confirmed in other bike studies on gender differences [81,82,83,84]. Women bicycle more slowly through intersections [85] and, because bicycle signals were associated with low risk of crash, bicycle signals could be installed that provide more time. The bicycle signals could have a red/green countdown number in the middle and red and green bicycles on the top and bottom, as in China, to give maximum information to the bicyclists and car drivers. 

### 4.3. If the Findings Are in Bicycle Facility Design Guidelines and CPTED Principles

The current AASHTO [27] and NACTO bicycle guidelines [59] detail how to build bicycle facilities but the schematics resemble engineering plans for building roads and lack consideration of human behavior perceptions, as identified by pioneers in the environment and behavior field of study [86,87,88,89]. These guidelines should have chapters that focus on the bike surface and the context. While Jane Jacobs was able to help stop urban renewal and lessen crime through “eyes on the street,” her vibrant neighborhood was primarily white upscale-chic and she focused on walking [62]. Crime Prevention Through Environmental Design (CPTED) focuses on crime, such as burglary, and stresses the importance of residents knowing their neighbors but people on a Main Street sidewalk or biking on a cycle track will be strangers. CPTED also focuses on pedestrians who have their feet on the ground while a bicyclist knocked from their bicycle can watch their bike disappear at speed. New CPTED principles should address bicycle environments following the perceptions of the most vulnerable, individuals in lower-income ethnically diverse communities.

### 4.4. Reflections on the Relevance and Implications of the Findings

Improving bike environments in lower-income ethnically diverse neighborhoods would increase biking in these populations but all built environment changes should now respond to Climate Change. If given cycle tracks, more individuals would bicycle [33,34,35,39,43,82,90] and, if bicycle and E-bike usage increases, mobile source air pollution could be reduced by 11% [91]. A cycle track/Bus Rapid Transit corridor with permeable surfaces and tree ditches would filter pollutants and foster tree growth, lessening heat island effect [92,93,94,95,96,97]. Wide cycle tracks could also serve as evacuation routes [98,99,100] because, in emergencies, non-functioning traffic lights and cars out of gas result in gridlock. Forceful advocacy is necessary to get cycle tracks built and the responsibility then rests with the lower income citizens. Rather than burden lower income residents with the unpaid time obligation of attending countless transportation hearings, wide cycle track networks should be justified throughout the city as a response to Climate Change. All of these cycle tracks should incorporate the design ideas of the lower income ethnically diverse residents because the concerns of the most fearful of crashes and crimes should come first. 

### 4.5. Limitations

There were 13 groups and only 212 total participants and this was not a random sample population. A representative sample of pictures of bicycle environments were included but more were not included due to time. Looking at the pictures on the large screen fostered discussions that might have biased the results because some may have been quiet. Limitations notwithstanding, there are several strengths including the populations sampled (community sense *n* = 116 and street sense *n* = 96) and their willingness to contribute their time and observations about the pictures. About half were female and 87% knew how to bike. The participants and community organizers chose the location for their survey and the food, enabling the participants to enjoy the process and understand that their comments were to change their neighborhood. 

## 5. Conclusions

During the 1950s and 60’s, construction of the U.S. Interstate Highway System was a form of slum-clearance and the roads were identified as “white men’s roads through black men’s homes.” [101] Some now suggest that “bike lanes are white lanes,” a term coined in Portland, Oregon when bike lanes were painted in an ethnic-minority neighborhood [102]. Yet, research in an ethnic-minority lower income community suggested the residents are biking and want cycle tracks [41]. While US DOT funding, under the banner of economic development, paid for demolishing what some considered slums and building highways, a new form of funding could spur economic development in ethnic-minority neighborhoods through the construction of cycle tracks designed based on the perceptions of residents. 

To lower crash risk, participants wanted wide two-way cycle tracks with surface color, bike stencils, arrows, and bicycle signals at the intersections. These features could help in wayfinding and lessen risk of crash or getting a ticket for unlawful biking. For lowering crime risk, participants wanted the wide cycle tracks to be on streets with high end stores, good sight lines, lighting, flowers, and limbed up trees. Though some have suggested bicyclists could use low stress routes/quiet side roads to lessen risk of crashing, those routes are isolated, making bicyclists in lower income neighborhoods vulnerable to crime. Instead, the cycle tracks in lower income/higher crime neighborhoods could be on Main streets where shops and cafes already exist and where more patrons would foster economic development. Rather than gentrifying the neighborhood, local residents could own and operate the shops and cafes. Because the current bicycle guidelines and CPTED principles do not include these lower-income ethnic-minority-identified environmental insights, perhaps the publication of new guidelines could help in the construction and location of bicycle facilities that serve all populations.

## Figures and Tables

**Table 1:** Bicycle environments in the 32 slides.

Facility	Description
(1) Road with no bicycle provision	Bicyclists are, by law, allowed to ride on all roads except interstate highways. The roads can be narrow one- or two-way neighborhood roads or multi-lane roads. The roads can also have parallel-parked cars.
(2) Road with sharrows (bicycle stencil and a double chevron)	A sharrow, shared lane marking, is to alert drivers that bicyclists will bicycle in that location in that travel lane. The sharrow also indicates to bicyclists the best position for riding within the lane. Sometimes, the sharrow is near the middle of the lane to avoid an opening car door.
(3) Painted bike lane	A painted bike lane is a portion of the roadway that can include a bicycle stencil and an arrow. A painted bike lane can also be beside parallel-parked cars or beside a curb. A bicycle lane has no physical separation to prevent drivers from driving into or parking in the space.
(4) One-way cycle track	A one-way cycle track has a barrier, such as bollards or parked cars, to prevent drivers from entering or parking within the cycle track. A cycle track is for the exclusive use by bicyclists and, unlike a shared use path, not shared with pedestrians. A one-way cycle track can be level with the road, level with the sidewalk, or travel through a park.
(5) Two-way cycle track	A two-way cycle track has a barrier, such as bollards or parked cars, to prevent drivers from entering or parking within the cycle track. The two-way cycle track has bicycle stencils and arrows with a line in the middle to indicate that bicyclists will be riding in two directions.
(6) Shared-use path	Shared use paths are asphalt/hard surface paths shared by walkers, bikers, joggers, in-line skaters, wheelchair users, baby carriage pushers, and scooters. These paths can be adjacent to roads, run through parks, or parallel property or waterways.

**Table 2:** Characteristics of community sense/street sense groups: age, sex, bike.

Area	Groups	N	Age Mean ± SDs (Min–Max)	Sex (Female, %)	Bicycle (Yes, %)
Total		212	36.6 ± 14.0 (18–79)	42.5	87.3
Commty. ^1^	1	18	33.9 ± 9.8 (18–53)	50.0	77.8
	2	21	45.8 ± 13.5 (22–66)	95.2	76.2
	3	11	31.3 ± 14.4 (18–65)	27.3	100
	4	14	40.8 ± 14.5 (18–60)	85.7	92.9
	5	16	32.6 ± 11.5 (21–52)	56.3	93.8
	6	10	29.8 ± 11.7 (18–46)	40.0	70.0
	7	17	46.8 ± 21.1 (19–79)	35.3	82.3
	8	9	38.7 ± 12.7 (22–57)	44.4	100
	All	116	38.3 ± 15.1 (18–79)	57.8	85.3
Street ^2^	9	14	34.5 ± 10.0 (22–60)	100	92.9
	10	21	32.0 ± 9.0 (18–52)	0	95.2
	11	13	36.6 ± 7.9 (25–54)	23.1	92.3
	12	20	21.9 ± 4.7 (18–38)	25.0	95.0
	13	28	44.9 ± 11.9 (25–67)	3.6	78.6
	All	96	34.5 ± 12.2 (18–67)	24.0	89.6

Note: ^1^ Strong community sense (church member, YMCA member); ^2^ Strong street sense (gang member, halfway house resident, homeless shelter resident).

**Table 3:** Summary statistics for perceptions of crime for each of the six bicycle facility types, stratified by sex, age, bicycle, and group type.

Bicycle Facility	All		One Way Cycle Track		Two Way Cycle Track		Road		Shared-Use Path		Bike Lane		Sharrow	
	Mean	Std	Mean	Std	Mean	Std	Mean	Std	Mean	Std	Mean	Std	Mean	Std
All	2.79	1.73	2.61	1.63	2.35	1.69	2.98	1.79	3.38	1.99	2.94	1.62	2.73	1.50
Sex														
Female	2.86	1.74	2.54	1.62	2.38	1.68	3.07	1.79	3.68	1.90	3.04	1.67	2.78	1.47
Male	2.75	1.72	2.67	1.64	2.33	1.69	2.91	1.78	3.14	2.02	2.85	1.57	2.69	1.53
*p* value	<0.0001 *		0.67		0.06		0.001 *		0.0007 *		0.001 *		0.37	
Age														
<25 years	2.84	1.76	2.72	1.65	2.44	1.74	3.03	1.79	3.38	2.04	2.92	1.65	2.73	1.61
25–34 years	2.80	1.74	2.60	1.62	2.29	1.69	3.07	1.77	3.32	2.03	2.95	1.64	2.77	1.53
35–44 years	2.74	1.68	2.59	1.57	2.28	1.56	2.85	1.85	3.40	1.93	2.92	1.49	2.70	1.43
45–54 years	2.69	1.89	2.41	1.84	2.28	1.85	2.97	1.88	3.09	2.20	2.87	1.83	2.71	1.58
≥55	2.88	1.55	2.68	1.45	2.49	1.59	2.92	1.58	3.76	1.62	3.06	1.47	2.71	1.29
*p* value	0.64		0.54		0.54		0.12		0.42		0.64		0.53	
Bicycle														
Can ride ^1^	2.76	1.71	2.59	1.60	2.37	1.67	2.92	1.76	3.39	1.98	2.89	1.59	2.67	1.48
Can’t ride ^2^	3.11	1.95	2.83	1.88	2.19	1.87	3.67	1.90	3.27	2.13	3.48	1.80	3.36	1.61
*p* value	<0.0001 *		0.04 *		0.23		<0.0001 *		0.17		0.001 *		0.007 *	
Group type														
Commty ^3^	2.62	1.64	2.42	1.55	2.21	1.55	2.73	1.71	3.42	1.91	2.72	1.53	2.58	1.39
Street ^4^	3.01	1.81	2.84	1.70	2.53	1.82	3.29	1.84	3.32	2.08	3.20	1.68	2.92	1.62
*p* value	<0.0001 *		<0.0001 *		0.0002 *		<0.0001 *		0.40		<0.00001 *		0.009 *	

Data are mean standard deviations, compared by using general linear models including age, sex, whether they know how to bicycle, and street sense or community sense, * *p* < 0.05; Score ranged from 0–6 (higher score = higher crime or crash = least preferred). ^1^ Know how to ride a bicycle; ^2^ Do not know how to ride a bicycle; ^3^ Strong community sense (church member, YMCA member), ^4^ Strong street sense (gang member, halfway house resident, homeless shelter resident).

**Table 4:** Summary statistics for perceptions of crash for each of the six bicycle facility types, stratified by sex, age, bicycle, and group type.

Bicycle Facility	All		One Way Cycle Track		Two Way Cycle Track		Road		Shared-Use Path		Bike Lane		Sharrow	
	Mean	Std	Mean	Std	Mean	Std	Mean	Std	Mean	Std	Mean	Std	Mean	Std
All	3.24	2.12	2.96	1.92	1.68	1.76	4.59	1.57	1.16	1.64	4.06	1.70	4.16	1.66
Sex														
Female	3.33	2.19	2.92	1.95	1.59	1.77	4.85	1.51	0.99	1.50	4.26	1.67	4.44	1.47
Male	3.17	2.06	2.99	1.90	1.75	1.76	4.38	1.59	1.30	1.73	3.89	1.72	3.95	1.77
*p* value	<0.0001 *		0.38		0.57		0.009 *		0.003 *		0.009 *		0.0008 *	
Age														
<25 years	3.24	2.11	2.97	1.88	1.81	1.88	4.59	1.55	1.09	1.76	3.98	1.70	4.11	1.61
25–34 years	3.26	2.10	3.00	1.91	1.68	1.69	4.59	1.52	1.09	1.59	4.11	1.73	4.07	1.67
35–44 years	3.20	2.18	2.96	2.03	1.37	1.64	4.66	1.59	1.14	1.60	4.08	1.61	4.18	1.79
45–54 years	3.22	2.14	2.82	1.91	1.69	1.81	4.48	1.71	1.46	1.81	4.05	1.83	4.27	1.60
≥55	3.30	2.03	2.97	1.83	1.95	1.75	4.57	1.54	1.17	1.35	4.05	1.70	4.33	1.64
*p* value	0.52		0.66		0.91		0.08		0.42		0.92		0.68	
Bicycle														
Can ride ^1^	3.23	2.11	2.96	1.91	1.68	1.74	4.57	1.56	1.12	1.60	4.04	1.71	4.10	1.68
Can’t ride ^2^	3.40	2.23	2.94	2.02	1.65	1.98	4.81	1.67	1.57	2.02	4.29	1.61	4.82	1.26
*p* value	0.18		0.79		0.78		0.54		0.02 *		0.59		0.08	
Group type														
Commty ^3^	3.12	2.07	2.75	1.82	1.52	1.67	4.53	1.56	1.17	1.58	3.91	1.66	4.05	1.62
Street ^4^	3.39	2.16	3.21	2.00	1.88	1.85	4.66	1.59	1.15	1.72	4.24	1.75	4.30	1.70
*p* value	<0.0001 *		0.009 *		0.002 *		0.0002 *		0.48		0.009 *		0.0004 *	

Data are mean standard deviations, compared by using general linear models including age, sex, whether they know how to bicycle, and street sense or community sense, * *p* < 0.05; Score ranged from 0–6 (higher score = higher crime or crash = least preferred). ^1^ Know how to ride a bicycle; ^2^ Do not know how to ride a bicycle; ^3^ Strong community sense (church member, YMCA member), ^4^ Strong street sense (gang member, halfway house resident, homeless shelter resident).

**Table 5:** Surface and Context Design Variables Related to Crime or Crime Perception.

Bicycle Facility Design Variable—Perception of Crime or Crash Times the Design Variable Was Mentioned during the 13 Group Sessions	One-Way Cycle Track	Two-Way Cycle Track	Road	Shared-Use Path	Bike Lane	Shar-row	Total
**Surface/bicyclist’s right-of-way—High Risk of Crime (bicyclist vulnerable)**							
Bad condition of road surface (potholes, uneven color, oil on road where cars stop at intersection—low vehicle maintenance, litter in street—old newspapers, etc.)		1	3				4
Narrowness of lane		2	2				4
Faded lines and symbols (bike stencil and arrows)		1			1		2
**Surface/bicyclist’s right-of-way—Low Risk of Crime (bicyclist less vulnerable)**							
Two-way cycle track so know how to get back home		2					2
**Surface/bicyclist’s right-of-way—High Risk Crash (more likely hit by vehicle)**							
No bike signs/stencils for bikers		1	16		1	1	19
Too narrow right-of-way			6		2	1	9
Sharrow nearer middle of road						4	4
Narrow cycle track with curbs on both sides	1	2					3
No section between parallel parked cars and cycle tracks for parked car doors to open	1				1		2
**Surface/bicyclist’s right-of-way—Low Risk Crash (less likely hit by vehicle)**							
Median for cycle track (raised island, delineator posts, diagonal paint lines, etc.)	16	7					23
Paint or color (red) designating location for bicyclists (lane, cycle track)	6	1			1	1	9
Wide right-of-way (road, cycle track, etc.)	2				2		4
Bike symbol stencil/arrows for bikers	3	1					4
One way cycle track	2						2
**Context of bicyclist—High Risk of Crime (bicyclist vulnerable)**							
Secluded/no people around (few people driving on wide streets)	2	8	5	12	2	1	30
Building types associated with crime (triple deckers, projects, solid walls, warehouses, closed storefronts, abandoned buildings, check cashier business, etc.)	3	4	10		9	1	27
Dark (little sunlight and no street lights or lights from houses)	3	10	1	6	4	1	25
Too many trees, bushes, dense foliage, high grass	1	8	4	6	3		22
Hiding spots, cuts (spaces between buildings)	1	8	3		6	1	19
Houses, front steps, balconies not painted or maintained, graffiti, dirty signs, telephone poles leaning, oil on road where parallel parked cars park (low maintenance on cars)	6		1		2		9
Too many people in crowds, tourist area	4	2	2		1		9
Side street (few cars, few eyes on street)	1	1	1	1	1		5
Narrow right-of-way or closed in		1	4				5
Only residential buildings or homes (everyone at work so no one sees crime)			2			1	3
McDonalds/fast food	3						3
Jumbled bike racks with too many bikes (can steal and not be noticed)	3						3
Low end stores and low end cars	1		1				2
Adjacent fence or knoll		1	1				2
Parallel parked cars (can hide between parked cars and also run away)	1				1		2
Lots of cars driving or parked	1				1		2
**Context of Bicyclist—Low Risk of Crime (bicyclist less vulnerable)**							
People out walking/driving and people watching from second story windows (see and report a crime)	6	7	6		3		22
Clean and nice signs, clean sidewalks	4	4	5		1		14
Open areas with good sight lines and not dark alleys	7	2	3		1		13
Balconies on houses, windows overlooking street from second story	3	4	1		2		10
A lot going on, sidewalk cafes with people sitting	4	2	1		1		8
Lots of light during day and at night	3	2	2	1			8
Commercial areas because have surveillance cameras	1	2	3		1		7
Mixed areas with residences and businesses	1	1	1		2		5
Flowers—nice plants		3	1		1		5
No trees or limbed up trees	1		3				4
High end stores and high end cars					2		2
Lots of little shops	2						2
**Context of bicyclist—High Risk of Crash (more likely hit by vehicle)**							
Drivers going in different directions around the bicyclists, don’t know direction of drivers	5		10		11	4	30
Close to cars in motion			11		10	3	24
Painted lane/cycle track—could get doored on both sides (driver exiting parallel parked car and passenger exiting car stopped in travel lane—van in lane)	4		2		13	1	20
Bike facility around bus stop, sharing road with buses, buses on both sides of bicyclist			9		9		18
Road encourages high speeds of vehicles, road rage, and drivers honking	1		4		3		8
Confusing paint	5		1		1		7
Able to be hit from behind			2		1	1	4
Too many intersection coming together	3						3
**Context of bicyclist—Low Risk of Crash (less likely hit by vehicle)**							
More people around to call in crash with cell phone, get license number, stop driver, witness crash, cameras	2	1	3		2		8
Not a lot of car traffic			2	1	1	1	5
High end cars on road		1	1		1		3
Bike signal at intersection	2						2
Few people out walking (peds get hit)		1				1	2
